# Chlorierte Biphenyle: MAK-Begründung, Nachtrag

**DOI:** 10.34865/mb0cbphpcbd10_1ad

**Published:** 2025-03-31

**Authors:** Andrea Hartwig, MAK Commission

**Affiliations:** 1 Institut für Angewandte Biowissenschaften. Abteilung Lebensmittelchemie und Toxikologie. Karlsruher Institut für Technologie (KIT) Adenauerring 20a, Geb. 50.41 76131 Karlsruhe Deutschland; 2 Ständige Senatskommission zur Prüfung gesundheitsschädlicher Arbeitsstoffe. Deutsche Forschungsgemeinschaft, Kennedyallee 40, 53175 Bonn, Deutschland. Weitere Informationen: Ständige Senatskommission zur Prüfung gesundheitsschädlicher Arbeitsstoffe | DFG

**Keywords:** Chlorierte Biphenyle, Entwicklungstoxizität, MAK-Wert, maximale Arbeitsplatzkonzentration, Schwangerschaftsgruppe

## Abstract

The German Senate Commission for the Investigation of Health Hazards of Chemical Compounds in the Work Area (MAK Commission) derived a concentration in workplace air for chlorinated biphenyls (PCBs) [1336-36-3] which is without risk to the developing foetus (corresponding to Pregnancy Risk Group C). The derivation is based on the prerequisite for the classification in Pregnancy Risk Group C at 3.5 µg/l plasma for the sum of the 6 indicator congeners (6IC) PCB 28, PCB 52, PCB 101, PCB 138, PCB 153 and PCB 180. Assuming a background concentration of 2 µg 6IC/l plasma for women up to 45 years of age with data from about 2010, an increment of 1.5 µg 6IC/l plasma is thus tolerable. This increment was estimated to correspond to a total PCB concentration in air of 0.0008 mg/m^3^ measured as the sum of the 6IC multiplied by 5. This concentration represents a rather conservative estimate, as the background concentration in plasma has reduced since and a respiratory volume of 10 m^3^ has been assumed, which might overpredict the exposure at workplaces in PCB-contaminated office or public buildings. Additionally, age and duration of exposure are important determinants for the inner exposure. These factors should be considered when evaluating the measurement results.

**Table:** 

**MAK-Wert (2015)**	**0,003 mg/m^3^ E^a)^**
**Spitzenbegrenzung (2015)**	**Kategorie II, Überschreitungsfaktor 8**

**Hautresorption (2012)**	**H**
**Sensibilisierende Wirkung**	**–**
**Krebserzeugende Wirkung (2015)**	**Kategorie 4**
**Fruchtschädigende Wirkung (2015)**	**Gruppe B^b)^**
**Keimzellmutagene Wirkung (2015)**	**Kategorie 5**

**BAT-Wert (2015)**	**15 µg/l Plasma für Σ PCB 28, PCB 52, PCB 101, PCB 138, PCB 153, PCB 180^c)^**
**BAR (2011)**	**PCB 28: 0,02 μg/l Plasma** **PCB 52: < 0,01 μg/l Plasma** **PCB 101: < 0,01 μg/l Plasma**
CAS-Nr.	1336-36-3

^a)^ (PCB 28 + PCB 52 + PCB 101 + PCB 138 + PCB 153 + PCB 180) × 5 entspricht Gesamt-PCB in Luft

^b)^ Voraussetzung für Zuordnung zu Schwangerschaftsgruppe C siehe „Bewertung“

^c)^ entspricht Gesamt-PCB im Plasma

## Voraussetzung für Zuordnung zu Schwangerschaftsgruppe C

In diesem Nachtrag wird die Konzentration in der Luft am Arbeitsplatz abgeleitet, bei der PCB der Schwangerschaftsgruppe C zugeordnet wären. 

Als Grundlage dafür dient die Ableitung der Plasmakonzentration von 3,5 µg PCB-Indikatorkongenere/l – basierend auf umfangreichen epidemiologischen Studien zu den empfindlichsten Endpunkten Entwicklungsneurotoxizität und Geburtsgewichtsverminderung sowie Tierdaten – bei der eine fruchtschädigende Wirkung nicht anzunehmen ist. Die Konzentration von 3,5 µg/l Plasma für die Summe der sechs Indikatorkongenere (6IK) (PCB 28, PCB 52, PCB 101, PCB 138, PCB 153 und PCB 180) gilt damit als Hinweis auf Voraussetzung für Schwangerschaftsgruppe C (Brinkmann et al. 2019).

Der MAK-Wert in Höhe von **0,003 mg Gesamt-PCB/m^3^**wurde anhand der Wirkung der dioxinähnlichen PCB festgelegt. Um auch die Wirkung der nicht-dioxinähnlichen PCB zu bewerten, wurde ausgehend vom NOAEL für PCB 153 von 17 µg/l Plasma für die 6IK eine Luftkonzentration berechnet. Dabei wurde die Hintergrundkonzentration von 8 µg Gesamt-PCB/l Blut (= 8 µg/l Plasma für die 6IK; Höchstwert der Hintergrundkonzentration von 1999) berücksichtigt. Eine zusätzliche Plasmakonzentration von 9 µg IK/l der nicht-dioxinähnlichen PCB sollte bei Einhaltung des MAK-Wertes nicht erreicht werden. Mit der Halbwertszeit von 12 Jahren für die **höherchlorierten** PCB (PCB 138, PCB 153 und PCB 180) wurde berechnet, dass 9 µg **höherchlorierte** IK/l Plasma einer Konzentration von 0,833 µg **höherchlorierte** IK/m^3^ entspricht (10 m^3^ pro Tag, 5 Tage/Woche, 100 % inhalative Resorption, 12 Jahre Halbwertszeit). Da jedoch in der Luft hauptsächlich niedrigchlorierte PCB mit geringerer Halbwertszeit vorhanden sind, die sich nicht so stark anreichern, schützt der MAK-Wert auch vor den Wirkungen der nicht-dioxinähnlichen PCB (Hartwig 2013). 

Der Hintergrundwert von 8 µg/l Plasma für die Summe der 6IK ist sehr konservativ, da sich seit 1999 die Hintergrundbelastung deutlich reduzierte: In einer Auswertung von Daten ab etwa dem Jahr 2010 betrug das 95. Perzentil der Hintergrundkonzentration für 36- bis 45-jährige Männer und Frauen 1,95 µg/l Plasma für die Summe der PCB 138, PCB 153 und PCB 180 (Tabelle 1). Ein bedeutsamer Geschlechtsunterschied wurde dabei nicht festgestellt (Schettgen et al. 2015).

**Tab. 1:** Plasmakonzentrationen [µg/l] von PCB in der erwachsenen Allgemeinbevölkerung in Deutschland (Schettgen et al. 2015)

**PCB-Kongener**	**Lagemaß**	**Alter [Jahre]**				
		**18–25**	**26–35**	**36–45**	**46–55**	**56–65**
		n = 157	n = 710	n = 400	n = 525	n = 357
PCB 138	Median	0,12	0,15	0,24	0,39	0,56
	95. Perzentil	0,25	0,33	0,53	0,93	1,26
	Maximalwert	3,1	0,71	1,08	1,70	3,98
PCB 153	Median	0,17	0,21	0,37	0,63	0,92
	95. Perzentil	0,38	0,49	0,79	1,41	1,94
	Maximalwert	0,84	0,89	1,55	2,65	5,45
PCB 180	Median	0,10	0,14	0,29	0,57	0,87
	95. Perzentil	0,29	0,34	0,65	1,23	1,87
	Maximalwert	0,40	0,77	1,43	4,59	9,08
PCB 138 + PCB 153 + PCB 180	Median	0,38	0,50	0,92	1,58	2,41
	95. Perzentil	0,88	1,14	**1,95**	3,54	4,82
	Maximalwert	1,80	2,37	3,57	8,19	18,5

Die Summe der Konzentrationen von PCB 138, PCB 153 und PCB 180 entspricht etwa der Konzentration der 6IK im Plasma bei nicht inhalativ mit PCB belasteten Personen (Tabelle 2 und 3), da bei diesen die anderen drei IK praktisch nicht nachweisbar sind.

Die inneren Belastungen bei Personen über 45 Jahren sind aber im Vergleich zu jüngeren Erwachsenen immer noch hoch. Diese Altersgruppe ist jedoch für die Bewertung der entwicklungstoxischen Wirkung nicht maßgeblich, da deren Anteil an der Gesamtgeburtenzahl mit 0,2 % extrem gering ist (im Jahr 2023: 1810 Geburten von Müttern über 45 Jahre (Statistisches Bundesamt 2024 b) bei insgesamt 692 989 Geburten in Deutschland (Statistisches Bundesamt 2024 a)). Deshalb wird für die Bewertung der entwicklungstoxischen Wirkung von der Hintergrundkonzentration der Altersgruppe 36 bis 45 Jahre von etwa 2 µg 6IK/l Plasma ausgegangen. Die erlaubte zusätzliche Belastung, um einen Spiegel von 3,5 µg 6IK/l Plasma nicht zu überschreiten, beträgt daher 1,5 µg 6IK/l Plasma (entspricht 3 µg Gesamt-PCB/l Plasma = 1,5 µg Gesamt-PCB/l Blut).

Bei inhalativer Exposition ist vorwiegend mit leichtflüchtigeren PCB in der Luft zu rechnen (ca. 95 % bei den 95. Perzentilen in Tabelle 2). 

**Tab. 2:** Konzentrationen der 6IK bei über die Luft Exponierten und bei Kontrollpersonen (Schettgen et al. 2012)

**Lagemaß**	**PCB-Kongener**						
	**28**	**52**	**101**	**138**	**153**	**180**	**6IK × 5**
	Konzentration in der Luft [ng/m^3^]						
Median	140	160	29	3	2	< 1	1740
95. Perzentil	320	348	86	22	13	2	3740
Max.	450	470	150	31	21	3	4280
	Konzentration im Blut der Exponierten [µg/l Plasma]						
Median	0,087	0,024	0,012	0,253	0,380	0,279	
95. Perzentil	0,352	0,091	0,046	0,846	1,256	1,085	
Max	0,878	0,426	0,124	2,226	3,360	3,179	
	Konzentration im Blut der Kontrollpersonen [µg/l Plasma]						
Median	< 0,01	< 0,01	< 0,01	0,263	0,392	0,301	
95. Perzentil	0,021	< 0,01	< 0,01	0,92	1,492	1,148	
Max.	0,059	0,029	0,015	2,437	3,523	3,186	

Die Halbwertszeiten der leichter flüchtigen PCB 28, PCB 52 und PCB 101 sind mit 2,4; 1,0 bzw. 1,3 Jahren deutlich geringer als die der hochchlorierten, wenig flüchtigen PCB mit zwölf Jahren (Esser et al. 2021). Nimmt man im Mittel zwei Jahre als Halbwertszeit für die leichter flüchtigen PCB an, kann die äußere Konzentration, um im Fließgleichgewicht (Ein-Kompartiment-Modell) 1,5 µg Gesamt-PCB/l Blut (= 1,5 µg 6IK/l Plasma) zu erreichen, wie folgt berechnet werden: 

1,5 µg Gesamt-PCB/l Blut × 300 (Fett : Blut-Verteilungskoeffizient) = 450 µg Gesamt-PCB/kg Fett, bei 20 % Körperfettanteil errechnet sich 90 µg Gesamt-PCB/kg KG. Bei einer Halbwertszeit von 2 Jahren (730 Tage) wird diese Konzentration bei täglicher Aufnahme von 85 ng/kg KG und Tag (ln2 × 90 µg/kg KG/730 Tage) erreicht. Unter Arbeitsplatzbedingungen (10 m^3^, 100 % inhalative Resorption, 70 kg KG, 5 Tage pro Woche) entspricht dies einer Konzentration von 0,833 µg Gesamt-PCB/m^3^ (85 ng/kg KG × 7/5 × 70 kg/10 m^3^). 

Wie schon oben ausgeführt, wird angenommen, dass die Zusatzbelastung nur durch leichtflüchtige PCB erfolgt, was auch weitgehend durch Messungen bestätigt wird (Tabelle 2 und 3).

In der Studie von Meyer et al. (2013) sind auch Daten für inhalativ belastete (im Median 12 Jahre exponiert in PCB-kontaminierten Wohnungen aus den 1970er Jahren; Expositionszeit unter der Woche 16 Stunden pro Tag; mediane Exposition im Jahr 2011: 0,000859 mg Gesamt-PCB/m^3^) und nicht belastete (PCB-Konzentration < Bestimmungsgrenze) Personen angegeben. Die Konzentration von 0,000859 mg Gesamt-PCB/m^3^ würde nach der obigen Berechnung zu einem Inkrement von ca. 1,55 µg 6IK/l Plasma (1,5 × 0,859/0,833) führen. Die inhalativ Exponierten hatten ein medianes IK-Inkrement von 1,91 µg 6IK/l Plasma. In der Berechnung wird angenommen, dass die Bewohner in einer Woche dasselbe Atemvolumen aufweisen wie bei 5-tägiger Arbeitsplatzexposition mit 10 m^3^/Tag. Tatsächlich dürfte die Belastung der Bewohner pro Woche höher sein, da auch eine häusliche Exposition am Wochenende anzunehmen ist. Es wird daher von einer Anwesenheit von 16 Stunden pro Tag an 7 Tagen der Woche bei einem Ruheatemvolumen von 10 m^3^ in 16 Stunden ausgegangen. Damit beträgt das vorhergesagte Inkrement der PCB-Belastung 7/5 × 1,55 µg 6IK/l Plasma = 2,17 µg 6IK/l Plasma.

**Tab. 3:** Konzentrationen der 6IK bei über die Luft Exponierten und bei Kontrollpersonen (Meyer et al. 2013)

**Lagemaß**	**PCB-Kongener**						
	**28**	**52**	**101**	**138**	**153**	**180**	**6IK × 5**
	Konzentration in der Luft [ng/m^3^]						
Median	61,4	94,6	8,9	0	0	0	859
	Konzentration im Blut der Exponierten [µg/l Plasma]						6IK
Median	1,371	0,216	0,034	0,157	0,392	0,341	2,715
	Konzentration im Blut der Kontrollpersonen [µg/l Plasma]						6IK
Median	0,014	< BG	< BG	0,134	0,346	0,262	0,805

BG: Bestimmungsgrenze

Die obigen Berechnungen des PCB-Inkrements stimmen also mit den Messergebnissen der Studie von Meyer et al. (2013) näherungsweise überein.

Allerdings stimmt die Beziehung zwischen äußerer und innerer Belastung in der Studie von Schettgen et al. (2012) (Exposition bei Arbeit in einem PCB-kontaminierten öffentlichen Gebäude, 209 Exponierte, 98 Kontrollpersonen) nicht mit der in der Studie von Meyer et al. (2013) überein (Tabelle 2). Hier war der Median der Luftkonzentration von Gesamt-PCB  von 1,74 µg/m^3^ fast doppelt so hoch wie bei Meyer et al. (2013), das Inkrement der Mediane für PCB 28 + PCB 52 + PCB 101 im Plasma war im Vergleich zu den Kontrollen jedoch nur 0,123 µg/l. Die drei höherchlorierten IK wurden nicht in die Inkrement-Berechnung einbezogen, weil die Kontrollpersonen höhere Werte aufwiesen als die Exponierten. 

Die oben durchgeführte Berechnung stimmt mit den Daten von Meyer et al. (2013) in etwa überein und überschätzt somit eher die PCB-Konzentration im Blut im Vergleich zu den Ergebnissen der Studie von Schettgen et al. (2012).

## Bewertung

Ausgehend von dem Hinweis für die Voraussetzung zur Zuordnung zu Schwangerschaftsgruppe C bei 3,5 µg/l Plasma für die 6IK PCB 28, PCB 52, PCB 101, PCB 138, PCB 153 und PCB 180 und einer Hintergrundkonzentration von 2 µg/l Plasma für die 6IK ist bei 0,0008 mg Gesamt-PCB/m^3^ (= 6IK × 5) eine Gefährdung der Schwangerschaft nicht anzunehmen. Bei höheren Konzentrationen ist aber nicht unbedingt auf eine Gefährdung der Schwangerschaft zu schließen, da die innere Belastung bei Stoffen mit sehr langer Halbwertszeit und altersabhängiger Hintergrundexposition wie PCB von der Expositionsdauer und auch dem Alter bei Beginn der Exposition abhängt. Das Fließgleichgewicht wird bei einer Halbwertszeit von 2 Jahren nach etwa 5 Halbwertszeiten, also 10 Jahren, erreicht. Bei kürzerer Expositionsdauer ist die innere Exposition entsprechend geringer. Die PCB-Konzentration im Blut ist ebenfalls geringer bei Personen unter 45 Jahren, da diese eine geringere Hintergrundbelastung aufweisen. Außerdem wurde mit einem Atemvolumen von 10 m^3^ für erhöhte körperliche Tätigkeit gerechnet, was nicht für alle Arbeitsplätze zutrifft, insbesondere nicht in PCB-belasteten Büroräumen. Weiterhin sind die Messungen vor ca. 15 Jahren erfolgt. In der Zwischenzeit sollten die Hintergrundwerte weiter abgesunken sein. Bei der Bewertung der Messdaten sollten diese Faktoren berücksichtigt werden.
